# Zika virus infection dysregulates human neural stem cell growth and inhibits differentiation into neuroprogenitor cells

**DOI:** 10.1038/cddis.2017.517

**Published:** 2017-10-12

**Authors:** Pradip Devhare, Keith Meyer, Robert Steele, Ratna B Ray, Ranjit Ray

**Affiliations:** 1 Department of Pathology, Saint Louis University, St. Louis, MO, USA; 2 Department of Internal Medicine, Saint Louis University, St. Louis, MO, USA

## Abstract

The current outbreak of Zika virus-associated diseases in South America and its threat to spread to other parts of the world has emerged as a global health emergency. A strong link between Zika virus and microcephaly exists, and the potential mechanisms associated with microcephaly are under intense investigation. In this study, we evaluated the effect of Zika virus infection of Asian and African lineages (PRVABC59 and MR766) in human neural stem cells (hNSCs). These two Zika virus strains displayed distinct infection pattern and growth rates in hNSCs. Zika virus MR766 strain increased serine 139 phosphorylation of histone H2AX (*γ*H2AX), a known early cellular response proteins to DNA damage. On the other hand, PRVABC59 strain upregulated serine 15 phosphorylation of p53, p21 and PUMA expression. MR766-infected cells displayed poly (ADP-ribose) polymerase (PARP) and caspase-3 cleavage. Interestingly, infection of hNSCs by both strains of Zika virus for 24 h, followed by incubation in astrocyte differentiation medium, induced rounding and cell death. However, astrocytes generated from hNSCs by incubation in differentiation medium when infected with Zika virus displayed minimal cytopathic effect at an early time point. Infected hNSCs incubated in astrocyte differentiating medium displayed PARP cleavage within 24–36 h. Together, these results showed that two distinct strains of Zika virus potentiate hNSC growth inhibition by different mechanisms, but both viruses strongly induce death in early differentiating neuroprogenitor cells even at a very low multiplicity of infection. Our observations demonstrate further mechanistic insights for impaired neuronal homeostasis during active Zika virus infection.

Zika virus infection and its association with microcephaly and Guillain–Barré syndrome have created an urgency to understand the disease mechanisms.^1, 2^ The probability of fetal microcephaly in Zika virus-infected pregnant women ranges from 1 to 13%. However, there is a great concern that other nervous system complications, although not as obvious as microcephaly, may be more prevalent.^3, 4, 5^ Microcephaly is most likely caused by loss (increased cell death) or differentiation failure of neuronal stem cells or their progenitor cell growth, impairing CNS development.^6^ Recently, Zika virus was identified in multiple locations in the USA, including New Jersey, New York and Texas. The virus has been detected in amniotic fluid of pregnant women and in the brain tissue of fetuses with microcephaly.^7, 8^

The structure of Zika virus is similar to other known flavivirus structures.^9^ Zika virus genome is a ~11 kb single-stranded, positive sense RNA that contains a single open reading frame. Once the RNA genome is released into the cytoplasm, it is directly translated into a polyprotein precursor. The polyprotein is cleaved by a combination of viral and host proteases to release three structural (C, prM and E) and seven nonstructural (NS1, NS2A, NS2B, NS3, NS4A, NS4B and NS5) proteins.^10^

The adult mammalian brain contains self-renewable, multipotent neural stem cells (NSCs) that are responsible for neurogenesis and plasticity.^11, 12, 13, 14, 15^ Extracellular matrix, vasculature, glial cells and other neurons are components of the niche where NSCs are located. This surrounding environment is the source of extrinsic signals that instruct NSCs to either self-renew or differentiate.^16^ NSCs give rise to neurons, astrocytes and oligodendrocytes. The African lineage of Zika virus (MR766) infects human induced pluripotent stem cell (hiPSC)-derived cortical human neuroprogenitor cells (hNPCs) *in vitro.*^17, 18, 19^ Zika virus infects multiple cell types within the developing brain and astrocyte infection may have an important role in initial infection by amplifying and distributing infectious virus to nearby neurons and glia.^20^ It was reported that Zika virus infection has a partial cytopathic phase characterized by cell rounding, pyknosis and caspase-3 activation.^21^ Despite notable cell death, Zika virus did not activate a cytokine response in hNPCs. This lack of cell intrinsic immunity to Zika virus is consistent with persistence of virus replication in hNPCs. A cellular RNA binding protein Musashi-1 (MSI1) has been shown to support Zika virus replication.^22, 23^ The developmental stage of neural progenitor cells is a determinant of the level of MSI1expression and neural progenitor cells are most susceptible to Zika virus when immature.

In this study, we examined mechanisms of Zika virus-mediated impairment of human NSCs (hNSC) differentiation and progenitor cell growth. Our results suggested African and Asian Zika virus strains utilize different mechanisms for hNSC growth inhibition. To our knowledge, this is the first report demonstrating Zika virus strains inducing hNSCs growth inhibition differently, although both virus strains cause immature neuroprogenitor cell death at an early stage of differentiation, indicating disruption of neuronal homeostasis.

## Results

### Infection of Zika virus PRVABC59 and MRV766 strains display distinct E glycoprotein localization and growth pattern in hNSCs

**Figure 1:**
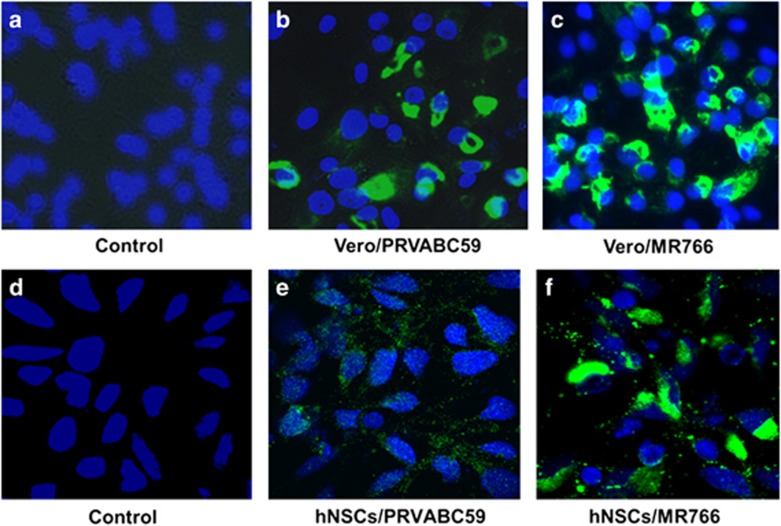
Subcellular localization of Zika virus E glycoprotein in virus-infected cells. Vero cells infected with PRVABC59 and MR766 displayed similar pattern of perinuclear and nuclear immunofluorescence of the E glycoprotein (**b** and **c**). Although hNSCs infected with PRVABC59 displayed localization of E glycoprotein primarily as perinuclear punctate dots, unlike MR766 (**e** and **f**). Mock-infected control (**a**,**d**) and virus-infected cell nuclei were stained with DAPI

hNSCs and African green monkey kidney epithelial (Vero) cells were infected separately with the African Zika virus strain (MR766) and Asian Zika virus strain (PRVABC59) at a multiplicity of infection (moi) of ~0.1. Representative immunofluorescence images at 3 days post-infection are shown (Figures 1a and f). Vero cells infected with PRVABC59 and MR766 displayed a similar pattern of immunofluorescence for the E glycoprotein, which is localized in the perinuclear and nuclear region (panels b and c). However, hNSCs infected with PRVABC59 displayed localization of E glycoprotein as perinuclear punctate dots (panel e), whereas MR766 E glycoprotein displayed a similar pattern of localization as observed in Vero cells (panel f). Our results suggested that PRVABC59 grows 5- to 10-fold higher in Vero cells (3 × 10^5^ ffu/ml) as compared with hNSCs (6 × 10^4^ ffu/ml), whereas no significant difference was observed in growth of the MR766 strain in these two cell lines when infected at a similar 0.1 moi. The growth of MR766 could be higher in hNSCs as this virus strain was adopted by several passages in cell culture, unlike PRVABC59.

### Zika virus infection in hNSCs induces DNA damage response

As Zika virus infection causes neuronal disease, we examined the status of cell regulatory genes following infection. We performed a cell cycle PCR array using Zika virus PRVABC59-infected hNSCs at day 3 post-infection or mock-infected cells to identify the molecular changes associated with Zika virus-mediated neuropathogenesis. We observed upregulation of several genes upon viral infection (Table 1). Genotoxic stress activated checkpoint complex (DNA damage response (DDR)) genes, especially HUS1 (23.64-fold), the 9-1-1 complex genes RAD1 (2.1-fold), RAD17 (2.1-fold) and MRE11A (3.4-fold) were upregulated in virus-infected cells when compared with mock-infected control cells. In addition, expression of genes associated with cell cycle arrest, such as CDKN1B (3.5-fold), CDKN2B (4.4-fold), GADD45A (2-fold) and WEE1 (2.5-fold) were enhanced in virus-infected hNSCs.

**Figure 2:**
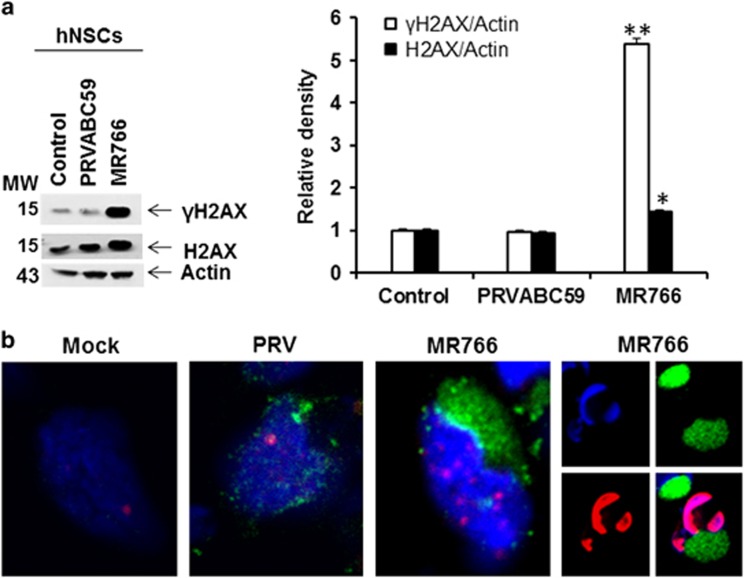
*γ*H2AX expression as DDR is significantly increased in MR766-infected hNSCs as compared with PRVABC59-infected cells. Western blot analysis for *γ*H2AX protein expression from Zika virus-infected hNSCs is shown (**a**). Blots were reprobed with total H2AX and actin-specific antibodies. Results of densitometric analysis are presented on the right. *P < 0.05, **P < 0.001. Confocal microscopy images from both virus-infected cells displaying localization of *γ*H2AX (red) and virus E protein (green) in merged image panels are shown (**b**). The nuclei were stained with DAPI (blue). Nuclear localization of distinct dots of *γ*H2AX is shown. hNSCs infected with MR766 also displayed higher expression of *γ*H2AX in nucleus with apoptotic nuclei (right panel)

As Zika virus infection enhanced HUS1 mRNA significantly, we next examined for DDR markers. One of these events is the phosphorylation of histone 2AX (H2AX).^24^ H2AX has a highly conserved serine residue at position 139 that is phosphorylated by ATM and/or ATR in response to DNA damage. ATM-dependent H2AX phosphorylation occurs in response to double-stranded DNA breaks. In contrast, ATR phosphorylates H2AX under circumstances of replication stress. *γ*H2AX is thought to amplify the DNA damage signal by enhancing and stabilizing the recruitment of DNA damage sensor proteins, such as ATR, ATM, Rad17 and the 9-1-1 complex (for which Hus1 is a component), and DNA repair proteins to the sites of DNA damage for repair. Interestingly, hNSCs infected with MR766 virus induced significantly higher *γ*H2AX and total H2AX expression (Figure 2a). A weak and similar basal level of *γ*H2AX expression in hNSCs and PRVABC59 virus-infected cells was observed. We also examined nuclear localization of *γ*H2AX by confocal microscopy. Immunofluorescence staining for phosphorylated H2AX, following Zika MR766 infection displayed characteristic focal pattern of *γ*H2AX known to be induced by DNA damage,^25, 26^ unlike mock control or PRVABC59-infected cells (Figure 2b). Some of the hNSCs infected with MR766 displayed strong *γ*H2AX nuclear staining with condensed nuclei (pyknosis) (Figure 2, panel b – right-hand photomicrographs) similar to earlier observations.^27^ MR766 grow more efficiently than PRVABC59 in hNSCs and could be a reason for higher *γ*H2AX expression.

Chk1 and Chk2 are transducer kinases for DDR signaling, which phosphorylate downstream molecules, including p53 and Cdc25 family proteins and control cell cycle arrest and apoptosis.^28, 29^ As *γ*H2AX was increased in Zika virus-infected cells, we investigated whether Zika virus activates the ATR-Chk1 or ATM-Chk2 signaling cascade. ATR phosphorylates Ser345 of Chk1 at stalled replication forks, whereas ATM phosphorylates the Thr68 of Chk2 upon DNA damage.^30^ p-Chk1 (Ser345) signal was not detected in Zika virus-infected hNSCs (Supplementary Figure 1, panel A). Chk1 cleavage because of genotoxic stress-mediated apoptosis was reported earlier^31, 32, 33^ and we observed cleaved immunoreactive Chk1 following Zika virus infection (especially MR766) in hNSCs. We also examined for p-Chk2 expression and was not detected in mock or Zika virus-infected hNSCs (Supplementary Figure 1, panel B). Reprobing of the same blot for total Chk2 showed an increase in Chk2 expression in PRVABC59-infected hNSCs, as compared with mock-infected cells. MR766-infected cells displayed faster migrating band suggesting an overall different regulation of endogenous Chk2 expression in Zika MR766 virus-infected hNSCs. Further, phospho-ATM/ATR expression was not observed in Zika virus-infected hNSCs. Our results suggested that Zika virus infection may utilize different DDR signaling mechanisms depending on the virus strain and needs further investigation.

**Figure 3:**
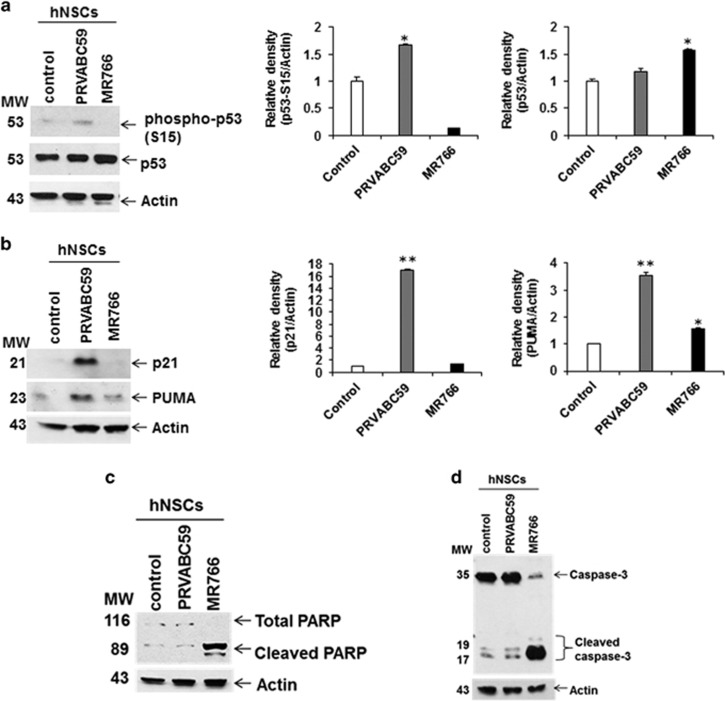
Zika virus-infected hNSCs display DDR response and promotes expression of cell cycle arrest and apoptotic markers. Western blot analysis for phospho-p53, p53, p21 and PUMA in PRVABC59 or MR766-infected hNSCs using specific antibodies (**a** and **b**). The blot was reprobed with an antibody to actin as an internal control. Densitometry analyses was performed from three experiments using ImageJ software and shown on the right. Data are represented as mean±S.D. Small bar indicates S.E. (*P<0.05; **P<0.01). PRVABC59 or MR766-infected hNSCs lysates were subjected to western blot analysis for PARP and caspase-3 using specific antibodies (**c** and **d**). The blot was reprobed with an antibody to actin as an internal control

The cellular DDR is a critical event for blocking cell proliferation and induction of apoptosis. We next examined the status of phospho-p53 (Ser15), a marker of p53 functional activation,^34^ in mock- or Zika virus-infected hNSCs. Results showed a modest increase in p53 Ser15 phosphorylation in PRVABC59-infected hNSCs as compared with mock-infected cells. However, MR766-infected cells exhibited reduced p53 phosphorylation (Figure 3a). Reprobing the same blot with total p53-specific antibody showed an increase of total p53 protein level in hNSCs infected with MR766 (Figure 3a). We also examined the downstream pathway of p53 signaling. We analyzed the expression levels of p53 targets p21 and PUMA in Zika virus-infected hNSCs. Interestingly, p21 was observed only in PRVABC59-infected hNSCs (Figure 3b). The expression of PUMA was also increased in PRVABC59-infected cells, whereas modestly upregulated in MR766-infected hNSCs although virus growth was significantly higher in comparison with PRVABC59. p21 is well-known cell cycle regulator and known to induce a permanent cell cycle arrest in response to DNA damage and p53 activation in most primary cells.^35^ Activated p53 may increase the expression of p21, which inhibits cyclin E-Cdk2 activity thereby inhibiting S-phase entry. Increased p53 activity following phosphorylation is known to induce expression of pro-apoptotic protein Puma that triggers cell death via regulation of mitochondrial permeability. Our results suggest hNSCs infected with PRVABC59 may promote p53-mediated signaling promoting cell cycle arrest.

To evaluate if induction of apoptosis occurs in both the lineages of Zika virus PRVABC59 and MR766-infected cells, we examined PARP cleavage, a characteristic hallmark of apoptotic responses, and induction of caspase-3. Cleavage of DNA repair enzyme PARP from a 116-kDa protein to a signature peptide of 86-kDa fragment is associated with a variety of apoptotic response. PARP is a nuclear protein and a downstream substrate of activated caspase-3/7. MR766 infection in hNSCs displayed cleavage of PARP and caspase-3 (Figures 3c and d). On the other hand, no significant induction of PARP or caspase-3 cleavage was observed in PRVABC59-infected hNSCs. Together these results suggest that PRVABC59 infection of hNSCs induces cell cycle arrest, whereas MR766 induces apoptotic cell death.

### Differentiating progenitor cells from Zika virus-infected hNSCs display high sensitivity for cell rounding and death

**Figure 4:**
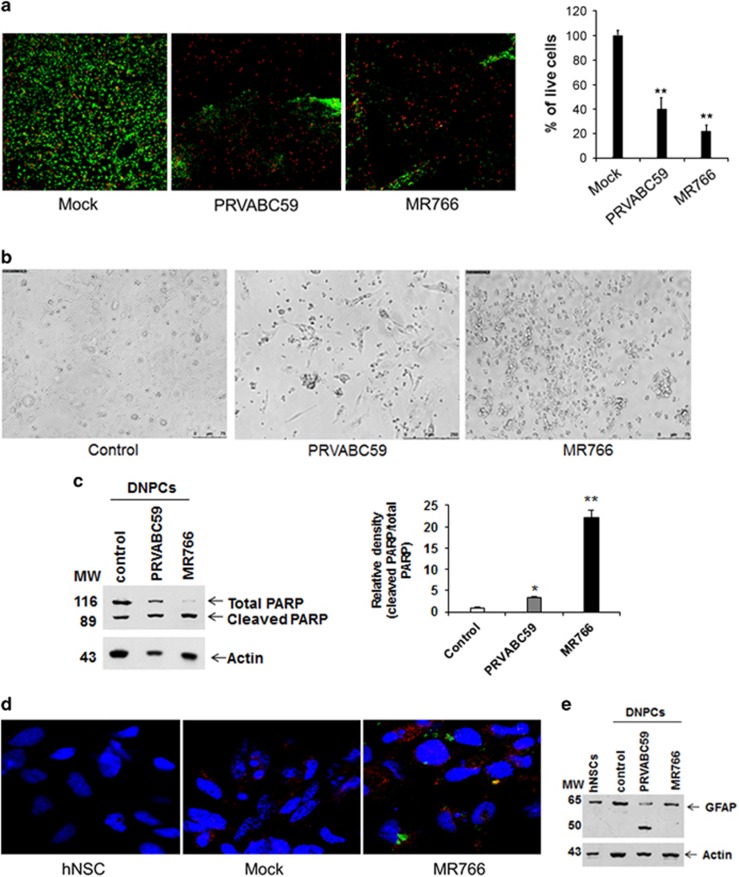
Cytopathic role of Zika virus infection in early differentiating neuroprogenitor cells. Mock-infected control, PRVABC59 and MR766-infected hNSCs were treated 24 h after infection with astrocyte differentiating medium for generation of neuroprogenitor cells. Cell viability assay of hNSCs infected with PRVABC59 and MR766 followed by incubation in differentiating medium for astrocytes are shown (**a**). Calcein AM stained live cells (green) and ethidium homodimer stained dead cells (red) are shown. A quantitation of live cells is shown on the right. Representative images of cytopathic effect after 48 h of incubation in differentiating medium from four independent experiments are shown (**b**). Western blot analysis was performed for analysis of PARP cleavage from Zika virus-infected cell lysates using specific antibody (**c**). The blot was reprobed with an antibody to actin as an internal control. Densitometry analyses was performed from three experiments using ImageJ software and shown on the right. Mock-infected or Zika virus-infected hNSCs incubated in differentiating medium for astrocyte generation were separately stained for GFAP marker (red) and viral E envelope glycoprotein (green) are shown (**d**), nuclei were stained with DAPI (blue color). We could not determine GFAP expression in PRVABC59-infected hNSCs for faster detachment from culture plate. Western blot analysis of GFAP was performed from mock, PRVABC59 or MR766-infected cell lysates using specific antibody (**e**). The blot was reprobed with an antibody to actin as an internal control. Data are represented as mean±SD. Small bar indicates standard error (*P<0.05, **P < 0.001)

We examined the effect of Zika virus on hNSCs differentiating into astrocyte-specific progenitor cells. hNSCs were grown in culture for 4 days. Cells were subsequently treated as mock-infected, infected with Zika virus PRVABC59 or MR766 strains at a moi of 0.02. Infected hNSCs were transferred into astrocyte-specific neuroprogenitor cell culture differentiation medium after 24 h of infection. Zika virus-infected differentiating progenitor cells were stained at an early time point (30 h) to examine differences in cell death as compared with mock-infected cells by calcein AM cell viability assay. Virus-infected cells exhibited a much higher number of dead cells before rounding and detachment from CellStart matrix-coated plate (Figure 4a).

Differentiating neuroprogenitor cells infected with both Zika virus strains displayed cell rounding and death within 36–48 h, whereas mock-treated cells did not exhibit any significant death during this time and appeared healthy. Representative images of phase contrast view are shown as illustrations (Figure 4b). Further incubation of mock-infected cells exhibited gradual appearance of astrocyte-like colonies around 9–12 days, and did not show any major sign of cell death or rounding similar to Zika virus-infected cells during this entire incubation period. Interestingly, a small number of differentiating progenitor cells infected with PRVABC59 strain exhibited elongated morphology, unlike MR766-infected cells. As we observed neuroprogenitor cell rounding following Zika virus infection, we next examined whether apoptosis is induced. Neuroprogenitor cells differentiated from hNSCs when incubated with either of the two Zika virus strains displayed a cleaved 86-kDa signature peptide of PARP (Figure 4c). Glial fibrillary acidic protein (GFAP) is the hallmark intermediate filament protein in astrocytes, a main type of glial cells in the central nervous system (CNS). Astrocytes use their GFAP-containing IF network as a signaling platform and a structural scaffold that coordinates the appropriate responses of astrocytes in health and disease.^36^ hNSCs in parental culture medium or upon incubation in astrocyte differentiating medium exhibited GFAP staining indicating the presence of progenitor cells (Figure 4d). Similar GFAP marker expression and Zika virus E glycoprotein expression were observed at much lower intensity in differentiating Zika virus MR766-infected cells. We could not examine PRVABC59-infected cells similarly as these cells detached at an early stage after treatment with differentiation medium. We therefore examined GFAP expression from Zika virus-infected differentiating into neuroprogenitor cells (both floating and adherent) by western blot analysis using specific antibody. Our results showed two polypeptides migrating as~65, and ~50 Kds in PRV-infected cells (Figure 4e). Interestingly, the higher molecular band (65 Kd) was present in mock-treated control hNSCs, mock-infected or infected differentiating progenitor cells with MR766. The lower molecular weight immunoreactive band (~50 Kd) was detected in PRVABC59-infected cell lysates, and the intensity of ~65 Kd band was much weaker as compared with the other lanes. Changes in GFAP expression and/or phosphorylation have been reported during brain damage or CNS degeneration.^37^ We speculate ~50 Kd band may represent differentially regulated GFAP and need further authentication. Although GFAP has several phosphorylation sites, very little is known about their modification following Zika virus infection, and will be studied in the future. Our results further suggest that different Zika virus strains follow distinct signaling pathways toward pathogenesis.

## Discussion

The results from this study elucidated the relationship between Zika virus infection, hNSCs differentiation and progenitor cell damage by the Asian and African virus strains of Zika virus-infected at a similar moi. We observed different cellular responses following infection of two Zika virus strains in hNSCs. MR766 strain replicates at higher levels, as compared with PRVABC59 strain. Further, MR766 induces phosphorylation of H2AX without phosphorylation of ATM/ATR-Chk1/Chk2 signaling and induces PARP cleavage. On the other hand, PRVABC59-infected hNSCs displayed p53 phosphorylation, induction of p21 and PUMA, implicating cell cycle arrest. A small group of p53 effector proteins were suggested to act as critical mediators of Zika virus-induced growth arrest and apoptosis in hNPCs.^38^

DNA damage-induced host cell apoptosis may limit viral replication, and some viral gene products actively suppress apoptosis. In other settings, DNA damage signaling may benefit the virus.^39^ This does not appear to be the case with the inhibition of Zika virus growth inhibition, rather a cause of neural cell death, at least with MR766. Both Zika virus strains induced distinct *γ*H2AX foci. However, marked phosphorylation of H2AX is observed during MR766 infection of hNSCs – the disease-relevant target cells. *γ*-H2AX was distributed in a diffuse nuclear pattern in several cells, distinct from the *γ*-H2AX foci typical of the response to PRVABC56 viral infection.

**Figure 5:**
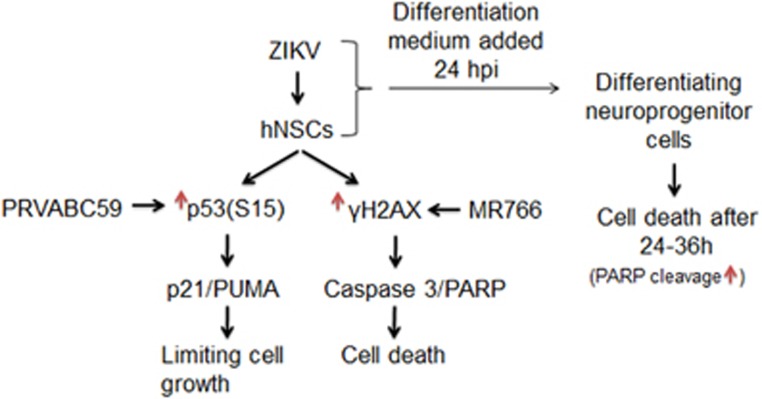
Summary of observations on neuronal damage by two different strains of Zika virus. hNSCs infected with PRVABC59 induced activation of the p53-p21 signaling axis, driving cells toward cell cycle arrest, whereas MR766-infected hNSCs were driven by apoptotic pathway. These cells infected with both Zika virus strains followed by incubation in astrocyte differentiating medium displayed cell death

In our study, we observed enhancement of p21 and PUMA expression in Zika virus PRVABC59-infected hNSCs (Figure 5). Zika virus PRVABC59-infected hNSCs displayed induction of the p53-p21 signaling pathway, suggesting promotion of cell cycle arrest. As p21 was reported to regulate self-renewal of NSCs,^40^ we postulate that PRVABC59-infected hNSCs are able to limit the DNA damage, which is in accordance with our findings of higher expression of p21 and low levels of *γ*H2AX, caspase-3 and PARP in PRVABC59-infected cells. On the other hand, MR766-infected hNSCs showed apoptotic cell death. It is important to note that hNSCs of different individuals may vary in neuronal differentiation potential following Zika virus infection^41^ but whether different strains of Zika virus affects neuronal differentiation differently will be an interesting aspect to explore further.

The literature relevant to RNA viruses and the DDR is emerging and focused mostly on HCV and the retroviruses.^42^ RNA viruses may have conflicting interactions with the DDR at several stages during their replicative cycles and can potentially inflict DNA damage through both direct and indirect mechanisms.^42^ Increased generation of ROS is a common feature of RNA virus infection and is a well-characterized source of endogenous DNA damage. RNA viruses can also acquire a survival advantage by targeting specific DDR proteins. Depletion of stem cells has been observed in mouse models defective for DDR components,^43, 44, 45, 46^ and may occur in Zika virus-infected hNSCs for impairment of self-renewal processes.^43^ The consequences of DNA damage processes for stem cells can be profound. Further understanding of the molecular mechanisms through which the DDR operates, in combination with the elucidation of the interactions between different DDR pathways may provide therapeutic opportunities for Zika virus-associated human diseases.

Interestingly, infection with both the virus strains promoted immature neuroprogenitor cell death and PARP cleavage even at a very low moi (0.001 or less). The possibility that cytokines in the viral inoculum may be responsible for the differing effects of MR766 and PRVABC59 on hNSCs. However, this seems to be unlikely as we generated both the viral stocks in Vero cells. If cytokine response is a determining factor for cell death, both the strains were expected to behave similarly in hNSCs. Instead, mechanisms for cell death were different. The role of Zika virus on differentiation of hNSCs into progenitor cells, and the underlying mechanisms for growth inhibition provide new insights into the potential damaging cellular response in the developing brain during infection.

We stimulated Zika virus-infected hNSCs for differentiation into progenitor cells. PRVABC59-infected cells displayed higher susceptibility to cell death as compared MR766-infected cells. Interestingly, much less Zika virus E protein of MR766 isolate is expressed in differentiating neuoprogenitor cells, indicating less virus replication as compared with parental hNSCs (compare between Figure 2b *versus* Figure 4e). This may explain, at least in part, virus pathogenesis and severity of disease primarily during fetal neurological development. Multiple variants of GFAP have been described in the literature.^47^ The GFAP gene consists of nine exons and eight introns and can be alternatively spliced to give rise to at least nine novel proteins. Changes in the expression level of the GFAP splice variants influence the intermediate filament network and may alter cell structure and mobility. GFAP can be phosphorylated at multiple sites including Thr-7, Ser-8, Ser-13, Ser-17, Ser34/38 and Ser-389. Phosphorylation of GFAP affects the formation of a stable intermediate filament network and remodeling of glial networks in mitosis. Interestingly, two Zika virus strains displayed variations in molecular sizes of GFAP. Understanding the phosphorylation status of GFAP in differentiated neuroprogenitor cells during Zika virus-associated microcephaly would be an important follow-up for further understanding neuronal disease progression. Further, we do not rule out the possibility of GFAP cleavage in Zika virus-infected cells as this smaller size band (~50 kDa) was observed only in PRVABC59-infected cells and will be interesting as a follow-up study in future.

In summary, our results uncovered potential mechanisms by which Zika virus induces genotoxic stress-mediated damage of not only hNSCs but also at the early developmental stage of neuroprogenitor cells. The observed differences between the two virus lineages (PRVABC59 and MR766) need further clarification from future studies.

## Materials and methods

### Virus stocks

Zika virus strains of African lineage (MR766, obtained from Robert Tesh, UTMB, Galveston, TX, USA), and Asian lineage (PRVABC59, Human/2015/Puerto Rico, obtained through BEI Resources, NIAID, Bethesda, MD, USA) were used for this work. MR766 is a highly cell culture adapted Zika virus strain than PRVABC59. Virus were grown in Vero cells, stocks were aliquoted, stored frozen and each aliquot thawed for single time use. Virus titter was determined by serial dilutions for infectivity in the same cell line and detected by immunofluorescence.

### Cell lines

hNSCs with proliferation and multipotent differentiation potential were purchased (Thermo Fisher Scientific, Waltham, MA, USA). hNSCs were derived from NIH-approved H9 (WA09) human embryonic stem cells (hESCs). Cells were maintained for proliferation in adherent cell culture when used with StemPro NSC SFM media following supplier’s instructions. Cells were maintained for differentiation into astrocytes in DMEM/F12 medium containing 2 mM l-glutamine, 1% penicillin–streptomycin and 10% FBS as described previously.^48^

### Immunofluorescence and confocal microscopy

Immunofluorescence assay was performed as described previously.^49, 50^ Briefly, hNSCs were seeded in a four-well chamber slide (Nunc) and remained mock or infected with Zika virus isolates PRVABC59 and MR766. Three days post-infection, the cells were washed with PBS, fixed with 3.7% formaldehyde for 20 min at room temperature and blocked with 3% bovine serum albumin for 1 h. The fixed cells were permeabilized with 0.2% Triton X-100 for 5 min at room temperature. Subsequently, the cells were incubated with Zika virus envelope protein-specific mouse antibody (BioFront, Tallahassee, FL, USA; BF-1176-56) and *γ*H2AX-specific rabbit antibody (Cell Signaling Technology, Danvers, MA, USA) overnight at 4 °C. The cells were washed and incubated with anti-mouse Ig conjugated with Alexa 488 and anti-rabbit Ig conjugated with Alexa 594 (Molecular Probes, Eugene, OR, USA) secondary antibodies for 1 h at room temperature. Finally, the cells were washed and mounted for confocal microscopy (Olympus FV1000, Waltham, MA, USA) after nuclei staining with DAPI. Images were superimposed digitally for fine comparisons.

GFAP immunostaining was done in differentiating astrocyte progenitor cells, control hNSCs or differentiating mock or MR766-infected cells. Cells were fixed after 48 h of differentiation media addition. The cells were stained for GFAP and Zika virus E protein-specific antibodies as mentioned above.

### Live/dead cell viability assay

Control or ZIKV-infected hNSCs undergoing differentiation were assayed for cell viability/death after 30 h of infection using Live/Dead two-color fluorescence assay according to the manufacturer’s instruction (Molecular Probes). Cells were washed in 1X DPBS and exposed to 4 *μ*m calcein AM and 2 *μ*M ethidium homodimer in DPBS for 30 min at room temperature. Dye uptake was determined under fluorescent microscope by observing green fluorescence (for calcein in live cells) and red fluorescence (for ethidium homodimer in dead cells).

### Real-time PCR array

RNA was extracted from mock or PRVABC59-infected hNSCs using TRIzol (Invitrogen, Carlsbad, CA, USA). A Human Cell Cycle specific PCR Array (PAHS-020ZA) (QIAGEN, Germantown, MD, USA) was performed as described previously.^51^ Array data were analyzed using free web-based software (http://pcrdataanalysis.sabiosciences.com/pcr/arrayanalysis.php) and automatically perform all ^ΔΔ^Ct fold change calculations.

### Western blot

Cell lysates were subjected to polyacrylamide gel electrophoresis and transferred onto a nitrocellulose membrane. Membranes were blocked with 5% nonfat dried milk and incubated with specific antibodies for *γ*H2AX (Ser139), H2AX, p-Chk1 (S345), Chk1, p-Chk2 (T68), Chk2, p-p53 (S15), p53, p21, PUMA, PARP and caspase-3 (Cell Signaling Technology). Rabbit monoclonal antibody produced by immunization with a synthetic peptide corresponding to residues surrounding Asp395 of human GFAP protein (Cell Signaling Technology). Proteins were detected by using enhanced chemiluminescence. The membrane was reprobed with actin as an internal control. The densitometric scanning of western blots was performed by ImageJ software (NIH, Bethesda, MD, USA).

### Statistical analysis

The results in the manuscript are presented as±S.D.. Data were analyzed by Student’s *t*-test with a two-tailed distribution. A *P*-value <0.05 was considered as statistically significant.

## Figures and Tables

**Table 1:** Gene upregulation in Zika virus-infected hNSCs as compared with mock-infected control cells by cell cycle array

**Signaling pathway**	**Genes**	**Fold upregulation**
DDR	HUS1	23.64
	MRE11A	3.4
	RAD1	2.15
	RAD17	2.12
	KPNA2	7.6
Cell cycle regulation	CDKN1B	3.5
	CDKN2B	4.4
	GADD45A	2.0
	AURKA	2.3
	E2F4	2
	WEE1	2.5
	SERTAD1	2.2
	STMN1	2.1
	CUL3	2.3
