# Supplementary material for: Zika virus infection dysregulates human neural stem cell growth and inhibits differentiation into neuroprogenitor cells
Source: Cell Death Dis. 2017 Oct 12;8(10):e3106–. doi: 10.1038/cddis.2017.517 (PMC5682681; doi:10.1038/cddis.2017.517)
Supplement: Supplementary Figure 1 [file cddis2017517x1.pdf]

Supplementary Materials

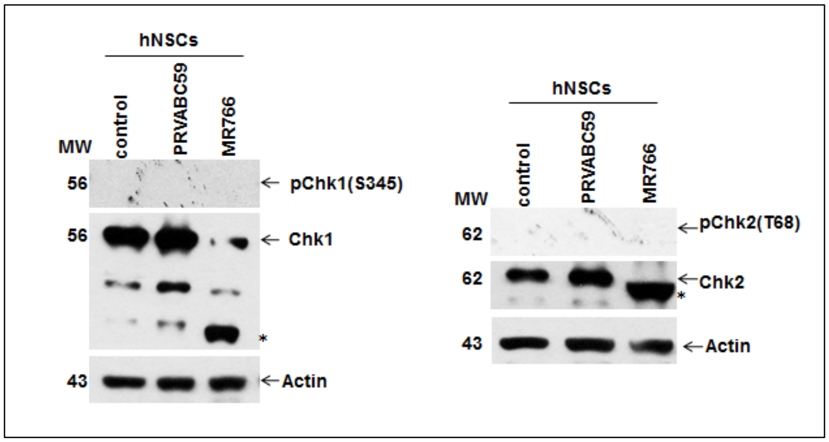

Figure 1 Chk1/Chk2 status in Zika virus infected hNSCs. Zika virus PRVABC59 or MR766 infected cells were analyzed for Chk1/Chk2 status by Western blot and compared with uninfected control cells. Phosphorylation status of Chk1 at Ser345 residue did not change, but total protein level modestly increased in PRVABC59 infected cells, and degraded in MR766 infected cells probably due to the apoptotic event (panel A). Zika virus PRVABC59 or MR766 infection did not induce phosphorylation of Chk2 at T68 residue, but enhanced total Chk2 protein expression, and degradation in MR766 infected cells (panel B). Higher mobility immunoreactive Chk1 and Chk2 bands are indicated by asterisks (*).
